# Circulating cell-free DNA use for diagnosing cholangiocarcinoma

**DOI:** 10.1186/s13148-019-0667-4

**Published:** 2019-05-14

**Authors:** Steven Sorscher

**Affiliations:** Oncology Division, Wake Forest School of Medicine, Winston-Salem, NC 27157 USA

To the Editor,

Currently, neither cell-free DNA (cfDNA) methylation assays nor next-generation sequencing (NGS) of circulating cfDNA are endorsed as methods to establish a diagnosis of cholangiocarcinoma or other malignancies. I agree with the conclusions by Wasenang et al. that their findings suggest that the application of serum cfDNA methylation assays offer a promising alternative to the more invasive methods currently considered necessary to secure a diagnosis of cholangiocarcinoma [1]. There is increasing evidence that NGS of circulating cfDNA appears to also be a less invasive alterative that may also be used to diagnose cholangiocarcinoma, an often difficult-to-diagnose malignancy.

Endoscopic retrograde cholangiopancreatography (ERCP) utilizes endoscopy to evaluate the biliary tract. In spite of ERCP with brushings (including forceps biopsies), ERCP with endoscopy, percutaneous cholangioscopy, and cholangioscopy with spyglass, diagnosing cholangiocarcinoma in patients suspected to have cholangiocarcinoma remains particularly challenging. For example, in 2018, a worldwide study revealed that 8–22% of patients “turned out” to have benign disease on microscopic examination of resected specimens [2].

In 2019, Mody et al. reported the largest series profiling the circulating tumor DNA (ctDNA) in patients with biliary tract tumors, which include cholangiocarcinomas and the closely related gallbladder cancers [3]. Aside from therapeutically relevant ctDNA alterations, they also noted that one or a number of other molecular alterations could be identified in the circulating DNA of these patients and Andersen and Jakobsen demonstrated that driver mutations in RAS and RAF seen in the tumors can typically be identified in cfDNA [3, 4]. Also, a patient with a molecularly diagnosed cholangiocarcinoma based on clinical suspicion and after multiple failed attempts at a tissue diagnosis has been described [5]. Identifying cfDNA with any of the oncogenic molecular abnormalities seen in the tumors of patients with known cholangiocarcinomas would be unexpected in patients without known underlying malignancies [6].

However, aside from being derived from a carcinoma from a different tissue of origin, there are tumor suppressor gene alterations described in cholangiocarcinomas which if found in cfDNA could very infrequently be related to a nonmalignant source. For example, identification of circulating BRCA mutated DNA might imply a germline BRCA mutation without an underlying related malignancy (particularly if the mutation allelic frequency is low) and circulating mutated TP53 might be related to clonal hematopoietic cells of indeterminate potential (CHIPs) [6].

However, it remains unclear whether particular cholangiocarcinomas (e.g., small tumors) shed enough cfDNA to identify an alteration associated with cholangiocarcinoma. Of note, Wasenang et al. demonstrated that “no significance difference in tumor size, stage, and survival time were observed between low and high methylation group” [1].

Neither cfDNA methylation assays nor NGS of circulating cfDNA is currently endorsed for diagnostic purposes [7]. The remarkable work by Wasenang et al. suggests that cell-free methylation of OPCML and HOXD9 assays could be useful in establishing a diagnosis of cholangiocarcinoma in patients suspected of having an underlying cholangiocarcinoma. Next-generation circulating cfDNA sequencing in patients suspected of having an underlying cholangiocarcinoma appears to be a promising and also minimally invasive tool as well to aid in diagnosing early-stage cholangiocarcinoma. Studies comparing these assays alone or in combination involving patients suspected—and before or later confirmed by tissue sample—to have cholangiocarcinoma will further clarify the role for these modalities in the early detection of this often difficult-to-diagnose disease.
